# Molecular genetic diagnosis of Glanzmann syndrome in Iranian population; reporting novel and recurrent mutations

**DOI:** 10.1186/s13023-019-1042-4

**Published:** 2019-04-27

**Authors:** F. Zafarghandi Motlagh, M. S. Fallah, H. Bagherian, T. Shirzadeh, S. Ghasri, S. Dabbagh, M. Jamali, Z. Salehi, M. Abiri, S. Zeinali

**Affiliations:** 1 Dr. Zeinali’s Medical Genetics Lab, Kawsar Human Genetics Research Center, No. 41 Majlesi St., Vali Asr St, Tehran, 1595645513 Iran; 2 Department of Biology, Pardis International, University of Guilan, Rasht, Iran; 3 Department of Medical Genetics and Molecular Biology, Faculty of Medicine, Iran University of Medical Sciences, Tehran, Iran; 4 Department of Molecular Medicine, Biotechnology Research Center, Pasteur Institute of Iran, Tehran, Iran

**Keywords:** Glanzmann thrombasthenia, α_IIb_β_3_, *ITGA2B*, *ITGB3*, Iran

## Abstract

**Background:**

Glanzmann thrombasthenia (GT) is a rare autosomal recessive abnormality of platelet aggregation with quantitative and/or qualitative abnormality of αIIbβ3 integrin. The αIIbβ3 is a platelet fibrinogen receptor, which is required for platelet aggregation, firm adhesion, and also spreading.

The disease is more prevalent in the populations with a higher rate of consanguineous marriages as in some Middle Eastern populations including Iraq, Jordan, and Iran. Different types of mutations in *ITGA2B* and *ITGB3* genes have been previously reported to cause the disease.

**Result:**

In this study, 16 patients with the clinical diagnosis of GT were studied. Direct sequencing of the exons and exon-intron boundaries of the above genes revealed mutations in 14 patients (detection rate: 87.5%). Briefly, out of fifteen types of identified mutations, 14 were novel. Seven mutations in the *ITGB3* gene included 4 missense [c.2T > C, c.155 G > T, c. 538 G > A, c.1990 G > T], one nonsense mutation [c.1303 G > T], a small deletion [c.1656_1658delCTC] and a deletion of one nucleotide [c.401delA]. Mutations in the *ITGA2B* were 8 different mutations consisting 2 missense [c.286 T > A, c.842 C > T], 2 deletions [c.1899 del T, c.189-319_236del], an insertion [c.1071_1072insG] and one splice site mutations [c.409–3 C > G], one synonymous mutation that might alter the normal splicing process [c.1392 A > G] and a nonsense mutation [c.1555 C > T].

The causative mutation in 2 patients remained unknown. Using long-range PCR and sequencing, we found a rather large deletion. The break point of this deletion covers 319 nt from the last part of the first intron and 48 nt from the beginning of the second exon of *ITGA2B* gene. The deletion was also detected in two unrelated patients with the same ethnicity. In addition, in silico analyses of novel mutations were performed.

**Conclusion:**

There was no recurrent mutation in the studied population. This may be due to either small sample size or the heterogeneity of the studied population.

## Introduction

Glanzmann thrombasthenia (GT, OMIM: 273800) is a rare inherited disorder of platelet function, caused by quantitative or qualitative defects of the platelet membrane glycoprotein (GP) IIb-IIIa (αIIbβ3) complex [1]. Patients have lifelong mucosal bleeding and may require platelet transfusions for severe bleeding episodes. Bleeding complications are also frequent after dental extraction, surgery, or delivery [2]. GT is a rare disorder worldwide but is relatively frequent in populations such as Iraqi, Jews, Jordanian Arabs, Iran, Palestine, South Indians and French Gypsies with a high frequency of consanguineous marriages [3, 4].

The molecular basis of GT is linked to quantitative and/or qualitative abnormalities of αIIbβ3 integrin that mediates binding of the adhesive proteins to attach aggregating platelets and ensure thrombus formation at sites of injury in blood vessels. GT is classified into 3 subtypes, depending on the level of αIIbβ3. Affected individuals show mucocutaneous bleeding with the absent of platelet aggregation in response to all physiologic stimuli. They show normal platelet count and morphology. In addition, platelet αIIbβ3 deficiency or dysfunction should always be confirmed.

Laboratory tests in severe GT show no platelet aggregation in response to all physiologic agonists and show reduced or absent clot reaction [5]. Patients with type I or classical GT have a virtual absence of αIIbβ3 complex (< 5% of normal). In type II GT, patients have up to 25% of the normal level of the complex. In the variant type, αIIbβ3 level is near normal but functionally impaired, leading to defective binding of fibrinogen [6, 7].

The αIIbβ3 complex integrin is encoded by *ITGA2B* and *ITGB3* genes (GPIIb and GPIIIa respectively). In brief, integrin synthesis occurs in the megakaryocytes with the αIIbβ3 complex formation in the endoplasmic reticulum (ER). The pre-GPIIb-IIIa complex is then transported to the Golgi apparatus, where sugar modification and cleavage of GPIIb into a heavy (M, 125,000) and light chain (M, 23,000) takes place [5]. GPIIb heavy and light chains remain covalently linked by a single disulfide bond. Following these processing events within the Golgi, the mature GPIIb-IIIa complex is then transported to the cell surface, where it exists in a resting state awaiting cellular activation. Noncomplexed or incorrectly folded gene products fail to undergo processing in the Golgi apparatus and are rapidly degraded intra-cellularly.

A continually updated mutation database is available on the *sinaicentral.mssm.edu/intranet/research/glanzmann* *website* [3, 4]. The molecular and functional characterization of the mutations has provided important insights to the better understanding of the biosynthesis and structure-function relationships of the αIIbβ3complex with the disease. It also adds important knowledge about the biology of other molecules of the integrin family [8, 9].

Genetic testing in GT patients is very important in the quality of life of the affected individuals and it facilitates prenatal diagnosis (PND) or pre-implantation genetic diagnosis (PGD) for at-risk families. The identified mutations will span the current database of the mutations of GT.

We aimed at identifying mutations underlying GT in Iranian patients from different parts of Iran. Most of the documented mutations were single nucleotide substitutions but large deletions are rarely reported. This study is reporting a novel large deletion of 367 bp in *ITGA2B* gene encompassing the last part of the first intron and the beginning part of the second exon of the gene.

## Material and methods

### Patients’ description

Sixteen unrelated families with the diagnosis of GT were investigated for the *ITGA2B* and the *ITGB3* genes mutations. The patients were referred to Medical Genetics Laboratory of Dr. Zeinali by hematologists. They were referred from the different parts of the country covering different races and ethnicities. Diagnosis of GT was based on the clinical picture of recurrent nasal hemorrhage, purpura, prolonged bleeding time (BT) and normal platelet count. Clinical features of each patient are reported in Table 1. Kawsar Human Genetics Research Center (KHGRC) ethical committee approved the research proposal.

**Table 1:** Mutations identified in this study, clinical features and disease outcome

Patients	Gene	Exon	Mutation	Protein	No. affected	Genotype	Relationship	Clinical features and laboratory tests	Family history	Reference
1	ITGB3	1	c.2T > C	p.Met1Thr	1	Homozygous	1st Cousin	Platelet Count, Bleeding Time:20 min Hematocrit: 35.5%	Yes	This study
2	ITGB3	10	c.1656_1658delCTC	p.Ser553del	1	Homozygous	1st Cousin	Platelet function test: Bleeding Time > 20 min, Prolonged Clotting Time	No	This study
3	ITGB3	4	c.401delA	p.Glu134GlyfsTer10	1	Homozygous	Not consanguineous but from the same village	Easy bruising, Hematemesis, Prolonged Clotting Time, Upper GI bleeding	No	This study
4	ITGB3	10	c.1303G > T	p.Glu435Ter	3	Homozygous	1st Cousin	Easy bruising, Menorrhagia, epistaxis, Purpura	Yes	This study
	ITGB3	2	c.155G > T	p.Cys52Phe	1	Compound Heterozygous	Non familial	Unknown	No	This study
5	ITGB3	4	c. 538G > A	p.Gly180Arg						
6	ITGB3	12	c.1990G > T	p.Glu664Ter	1	Homozygous	1st Cousin	Platelet function test: Bleeding Time: 17 min, Prolonged Clotting Time, Upper GI bleeding, Menorrhagia, epistaxis	No	This study
7	ITGA2B	19	c.1899delT	p.Cys633TrpfsTer17	2	Homozygous	1st Cousin	Bleeding Time > 20 min, Prolonged Clotting Time, Easy bruising, Menorrhagia, epistaxis	No 2 brothers	This study
8	ITGA2B	4	c.409-3C > G	intronic mutation with no amino acid change.	1	Homozygous	2nd Cousin once removed	Unknown	No	This study
9	ITGA2B	2	c.189-319_236del	–	2 Unrelated families	Homozygous	1st Cousin	Easy bruising, Menorrhagia, epistaxis, Purpura	Yes	This study
10	ITGA2B	13	c.1392A > G	p.Pro464Pro	1	Homozygous	1st Cousin	Platelet function test: Bleeding Time > 20 min, Prolonged Clotting Time Hematocrit: 38.1%	No 2 sisters	This study
11	ITGA2B	2	c.286T > A	p.Cys96Ser	1	Homozygous	1st Cousin	Abnormal Platelet aggregation, Bleeding time > 12 min, Clot retraction:25 min	Yes	This study
12	ITGA2B	8	c.842C > T	p.Thr281Ile	1	Homozygous	1st Cousin	Platelet function test: Bleeding Time > 20 min, Prolonged Clotting Time, Hematocrit: 39.6%	No	This study
13	ITGA2B	16	c.1555C > T	p.Gln519Ter	1	Homozygous	1st Cousin	Unknown	No	This study
14	ITGA2B	12	c.1071_1072insG	p.Arg358AlafsTer47	1	Homozygous	1st Cousin	Unknown	No	This study

Genetic counseling was performed and all participants signed informed consent forms. 10 ml of peripheral blood of the patients and their parents were provided in tubes containing EDTA as an anticoagulant. Genomic DNA were extracted by salting out method [10].

### Molecular genetic study

Genetic study of the patients was conducted using direct sequencing of the entire coding and exon-intron boundaries of the *ITGA2B* and the *ITGB3* genes [3, 4]. Primer sequences are available upon request. PCR amplification was performed in a total volume of 25 μl containing 2.5 μl of 10X buffer (Kawsar Biotech Co., Tehran, Iran, KBC), 2.5 μl DMSO (Sigma-Aldrich, USA), 0.96 mM dNTPs (KBC), 4 mM MgCl2 (KBC), 6 pmol of forward and reverse primers, 1 U Taq polymerase (KBC), and 30 ng of genomic DNA as a template. The thermocycling consisted of 1 cycle of initial denaturation at 95 °C for 5 min, followed by 30 cycles of 1 min denaturation at 95 °C, 1 min annealing at 64 °C, and 1 min extension at 72 °C, following a final extension at 72 °C for 10 min and storing the PCR product in 4 °C.

Sequencing.

PCR products were directly sequenced using BigDye Terminator kit (Thermo Fisher Scientific, USA, TF) according to the manufacturer’s protocol. Then the samples were run on an ABI3130XL Genetic Analyzer (TF). Sequences were compared with human genomic and cDNA sequences of the genes with the accession numbers of *ITGA2B* (NM: 000419, NC:000017) and *ITGB3* (NM:000212, NC:000017). Mutations nomenclature was conducted according to the recommendations of Human Genome Variation Society [11].

### Characterization of a deletion breakpoint

Systematic failure to amplify the 2nd and 3rd exons (since these exons are amplified together) of the *ITGA2B* suggested deletion of these two exons. To exactly determine the breakpoint, PCR- amplification with forward (F) of 2nd exon (2–3) and reverse (R) of 4th exon (3–4) was carried out. Primer sequences are listed below:

*ITGA2B* (2–3) F: GTCTGTGAGGTGTCATTGAGGA.

*ITGA2B* (3–4) R: GCTTCACAGTAACGCTTGTCC.

To find out the break point of this deletion, primer walking and subsequent sequencing were applied. The reaction mixture and PCR parameters were as above.

In order to check the frequency of novel missense variants, we performed ARMS-PCR in 100 ethnically matched healthy controls. Sequences of ARMS primers for these tests can be provided upon request. Bioinformatics analysis was also performed to elucidate the clinical significance of the identified variants. We used available online bioinformatics tools such as SIFT and PolyPhen Project to see the impact of missense variants on disease pathogenicity. Also, Mutation Taster was used for deletion and insertion variants [12].

## Results

The study included sixteen unrelated families with the diagnosis of GT. As it was expected most of the patients were the result of consanguineous marriages (14/16 families, 87%). Sequencing analysis revealed totally 15 different mutations in the *ITGB3* and the *ITGA2B* genes (Table 1). All identified mutations were homozygous except one in the *ITGB3*, which was in the form of compound heterozygous. Mutations in the *ITGB3* gene consist 7 different mutations including 4 missense mutations [c.2T > C, c.155 G > T, c. 538 G > A, c.1990 G > T], a nonsense mutation [c.1303 G > T], one small 3 nt deletion [c.1656_1658delCTC] and a deletion of a single nt [c.401delA]. Interestingly all identified mutations in this gene were novel (Table 1).

Mutation scanning in the *ITGA2B* revealed 8 different mutations including an insertion [c.1071_1072insG], 2 missense mutations [c.286 T > A, c.842 C > T], a nonsense mutation [c.1555 C > T], 2 deletions [c.189-319_236del, c.1899 del T], one splice site mutation [c.409–3 C > G] and one synonymous mutation that might alter the normal splicing process [c.1392 A > G].

The identified variants were heterozygote in parents and were not present as homozygote in their healthy members of the family as expected in cases where mutations are deleterious. To further confirm the pathogenicity of the missense variants, we checked the mutation in 100 random people and they were normal which supports the pathogenicity of the novel variants.

**Fig. 1:**
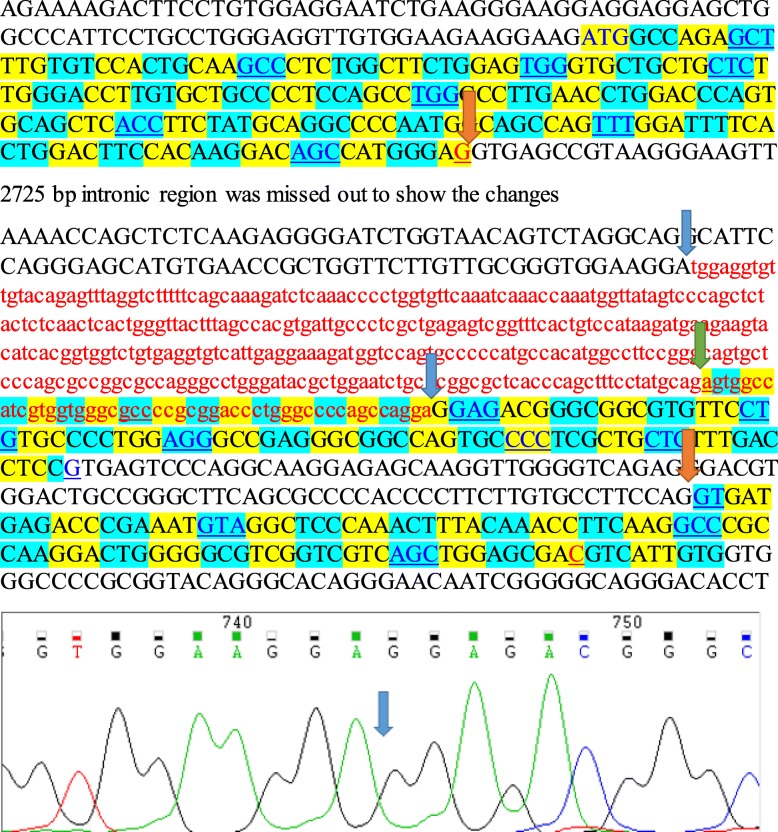
The figure shows schematic representation of the DNA sequences from the joining parts of the first and third exons. If this is the case, then AG nucleotides from the first exon and the G from the third exon will join together. [The orange arrows show the expected joining between exon1 and 3, which leads to a frameshift and a truncated protein. Lowercase red letters indicates the deleted part. The upper letters in black color show the intronic regions and upper letters in yellow and blue boxes show the exonic regions. Blue arrows show the start and the end of the deleted part. The green arrow indicates the first nucleotide at the beginning part of the second exon]

In a family, while sequencing exons 2 and 3, we could not get any band in the patients suggesting that theses exons may be deleted. In order to characterize the deletion breakpoints, primer walking and sequencing were performed. While the size of DNA fragment in normal cases was nearly 1500 bp as expected but the size of DNA product from the patient was around 1200 bp. Subsequent sequencing of this fragment confirmed deletion of 367 bp covering 319 nt from intron 1 and 48 nt in the second exon (Fig. 1).

## Discussion

GT is a rare congenital bleeding syndrome, which results in deficient platelet aggregation due to the absence or dysfunction of the fibrinogen receptor αIIbβ3 [12]. However, GT is rare worldwide but is relatively frequent in inbred populations such as Iraqi-Jews, Indians, and Arabs in Jordan, Israel, and Saudi Arabia. The exact number of GT patients in Iran has not been clearly reported. But it seems that the disease incidence will be higher from the estimated 1 in 200,000 in the US population. Because Iran is a country with nearly 38.4% rate of consanguineous marriage and at the other hand autosomal recessive mode of inheritance of the disease will add to the number of patients [13]. To the best of our knowledge, this study is the first comprehensive study on the molecular aspects of GT in Iranian population. Needless to say there is another similar study carried out by Kazemi et al. [14] on Iranian population but only three deleterious mutations have been reported out of 20 families affected with GT. However, none of the mutations reported by them is repeated in our study.

In Table 1 we have provided types of mutations and their effects at the amino acid level. Further in silico analysis was performed (Table 2) to find how these changes can be pathogenic. Obvious ones will not be discussed.

**Table 2:** Predicted pathogenicity of 12 novel mutations identified in this study

Gene name	Mutation at nucleotide level	Protein	SIFT	Polyphen	Mutation taster	HSF	(HumVar results)
ITGB3	c.2 T > C	p.Met1Thr	AFFECT PROTEIN FUNCTION with a score of 0.00	BENIGN with a score of 0.000	-disease causing -no abrogation of potential splice sites	No significant splicing motif alteration detected	BENIGN with a score of 0.000
ITGB3	c.1656-1658delCTC	p.Ser553del	-	-	-disease causing -splice site changes	-	-
ITGB3	c.1303 G > T	p.Glu435Ter	Affect protein function	-	-	Alteration of an exonic ESE site. Potential alteration of splicing.	-
ITGB3	c.155 G > T	p.Cys52Phe	AFFECT PROTEIN FUNCTION with a score of 0.00	This mutation is predicted to be PROBABLY DAMAGING with a score of 1.000	disease causing splice site changes	Creation of an exonic ESS site. Potential alteration of splicing.	This mutation is predicted to be PROBABLY DAMAGING with a score of 1.000
ITGB3	c. 538 G > A	p.Gly180Arg	AFFECT PROTEIN FUNCTION with a score of 0.00	This mutation is predicted to be PROBABLY DAMAGING with a score of 1.000	disease causing splice site changes	Activation of an exonic cryptic acceptor site, with presence of one or more cryptic branch point(s). Potential alteration of splicing.	This mutation is predicted to be PROBABLY DAMAGING with a score of 1.000
ITGB3	c.401 del A	p.Glu134GlyfsTer10	–	–	-disease causing -no abrogation of potential splice sites	Alteration of an intronic ESS site. Probably no impact on splicing	–
ITGA2B	c.1899 del T	p.Cys633TrpfsTer17	-	-	amino acid sequence changed frameshift protein features might be affected	Creation of an exonic ESS site. Potential alteration of splicing.	-
ITGA2B	c.409–3 C > G	–	-	-	-	Alteration of the WT acceptor site, most probably affecting splicing.	-
ITGA2B	c.1392 A > G	p.Pro464Pro	-	-	-	Alteration of the WT donor site, most probably affecting splicing. Alteration of an exonic ESE site. Potential alteration of splicing	-
ITGA2B	c.286 T > A	p.Cys96Ser	AFFECT PROTEIN FUNCTION with a score of 0.00.	PROBABLY DAMAGING with a score of 1.000 (sensitivity: 0.00; specificity: 1.00)	disease causing -splice site changes	Alteration of an exonic ESE site. Potential alteration of splicing.	PROBABLY DAMAGING with a score of 0.997 (sensitivity: 0.27; specificity: 0.98)
ITGA2B	c.842 C > T	p.Thr281Ile	TOLERATED with a score of 0.44	BENIGN with a score of 0.241 (sensitivity: 0.91; specificity: 0.88)	-disease causing amino acid sequence changed protein features (might be) affected	Alteration of an exonic ESE site. Potential alteration of splicing.	This mutation is predicted to be BENIGN
ITGA2B	c.1071-1072insG	p.Arg358AlafsTer47	-	-	disease causing amino acid sequence changed frameshift protein features (might be) affected splice site changes	-	-

The first patient (Table 1) with c.2T > C point mutation which is located at the very beginning of the first exon of the *ITGB3* gene (β3 subunit,) will change the methionine to threonine. Initiator methionine is very important for the initiation of protein synthesis.

Regarding the third patient with c.401delA mutation in the *ITGB3*, and based on mutation taster prediction [15] it is disease causing change. The fourth patient with c.1303G > T [p. Glu435Ter] mutation, causes change of glutamic acid to a termination codon leading to premature stop codon with only 434 residues which most probable will not function properly. The other patient had c.155 G > T mutation in the *ITGB3* gene, changing cysteine into phenylalanine. The wild-type residue is involved in a cysteine bridge formation, which is important for the stability of the protein. Only cysteine can make these types of bonds, so the mutation causes loss of this interaction and will have a severe effect on the 3D-structure of the protein. Together with the loss of the cysteine bond, the differences between old and new residue can cause destabilization of the structure [16], This prediction is also supported by SIFT and Polyphen results. The c. 538 G > A [p. Gly180Arg] mutation in the *ITGB3* changes glycine into arginine and introduces an amino acid with different properties that can disturb this domain and abolish its function. Because the wild-type residue, glycine, is the most flexible of all residues. Mutation of this glycine can abolish this function. This flexibility might be necessary for the protein’s function and by this mutation the torsion angles for this residue are unusual [16]. This prediction is also compatible with the SIFT and Polyphen prediction (Table 2).

The deletion of T nucleotide in exon 19 of *ITGA2B* gene (i.e. c.1899 del T [p. Cys633TrpfsTer17]) causes a frameshift in protein structure. HSF (Human splice finder) predicted that this frameshift created an exonic ESS site that has a potential to alter the splicing [17]. Regarding c.409–3 C > G and c.1392 A > G mutations in the *ITGA2B* which occurred near the potential splice sites of the protein, this can alter the wild type acceptor and donor site respectively. The c.189-319_236del mutation at *ITGA2B* gene leads to the elimination later part of the intron one and the beginning part of second exon. This deletion, therefore, deletes the splice acceptor site. This in turn should cause deletion of the entire exon 2 because there is no alternative splice site within the remaining part of exon 2 and therefore the remaining part of this exon will be eliminated via splicing of intron 1 (donor site), joining of exon1 and exon3**.** Therefore it is expected that through this splicing the exon 1 will be joined with exon 3. Figure 1 shows that if this would be the case then amino acids 63rd to 103rd will be lost and a frameshift would be resulted. The frameshift mutation leads to a truncated protein which loss of function would be resulted. Since we could not obtain fresh blood from the patient to perform RNA analysis, the above conclusion can be provided. However, whatever the consequence at the molecular level, we can claim that resulting effect at the protein level is having damaging effect on the patient.

It should be added that the identified variants were not present in the single nucleotide polymorphism database (Exome Variant Server, http://evs.gs.washington.edu/EVS/).

Regarding phenotype-genotype correlation, more studies with larger sample size is recommended.

It is interesting to see so many novel mutations in a rather small number of families (14/16) indicating several independent founder mutations. If we combine other mutations reported by others (Kazemi et al.) with ours it might become even more interesting. This may mean that either these genes are highly mutable (which may not be true since the disease is not so prevalent), or Iranian population is so diverse. This diversity has many historical proofs, which are beyond the scope of this paper but it worth mentioning.

## Conclusions

In conclusion, our investigation of 16 patients, and in some cases including their families, revealed a number of different mutations within the αIIb and β3 subunits associated with causing GT. This vast number of different mutations in different provinces of Iran indicates the genetic heterogeneity in the studied group as well as in GT in general. Further investigation on the prevalence of each mutation from each ethnicity may reveal different picture.

## Abbreviations

bp: Base pair
BT: Bleeding time
del: Deletion
EDTA: Ethylenediaminetetraacetic acid
ER: Endoplasmic reticulum
GP: Glycoprotein
GT: Glanzmann thrombasthenia
ins: Insertion
KHGRC: Kawsar Human Genetics Research Center
Nt: Nucleotide
PCR: Polymerase chain reaction
PGD: Pre-implantation genetic diagnosis
PND: Prenatal diagnosis
U: Unit; ng: nanogram
